# Single implant-supported two-unit cantilever fixed partial dentures in the posterior region: a retrospective case series with a mean follow-up of 6.5 years

**DOI:** 10.1186/s40729-021-00361-8

**Published:** 2021-08-19

**Authors:** Charlotte Jensen-Louwerse, Harjan Sikma, Marco S. Cune, Felix L. Guljé, Henny J. A. Meijer

**Affiliations:** 1 Center for Dentistry and Oral Hygiene, Dental School, University Medical Center Groningen, University of Groningen, PO Box 196, NL-9700AD Groningen, The Netherlands; 2 Department of Oral and Maxillofacial Surgery, Prosthodontics and Special Dental Care, St. Antonius Hospital, Nieuwegein, The Netherlands; 3 Department of Oral and Maxillofacial Surgery, Prosthodontics and Special Dental Care, University Medical Center Utrecht, Utrecht, The Netherlands; 4 Department of Oral and Maxillofacial Surgery, University Medical Center Groningen, University of Groningen, Groningen, The Netherlands; 5 Center for Dental Implants De Mondhoek, Apeldoorn, The Netherlands

**Keywords:** Implant-supported fixed partial denture, Cantilever, Dental implants, Posterior region

## Abstract

**Background:**

The aim of this retrospective study was to evaluate the implant survival, clinical and radiographic outcomes, and patient satisfaction of single implant-supported two-unit cantilever fixed partial dentures in the posterior region.

**Methods:**

Patients who received a single implant-supported fixed partial denture with a cantilever in the posterior region between January 2004 and February 2018 were included. Survival rate of the implants and the fixed partial dentures and data regarding the marginal bone level, presence of plaque, calculus, bleeding on probing, mucosa health, pocket probing depth, and patient satisfaction were collected during an evaluation visit. Complications were recorded from the medical records.

**Results:**

Twenty-three patients (mean age 64 ± 13 years) with 28 implants could be included in the study. The mean follow-up period was 6.5 ± 4.8 years at the time of data collection. The survival rate of the implants and fixed partial dentures was 100%. Mean marginal bone loss for the mesial and distal side of the implants was 0.41 mm (SD 1.18 mm) and 0.63 mm (SD 0.98 mm) respectively. A high prevalence of peri-implant-mucositis (89.3%) and peri-implantitis (17.9%) was observed as well as a limited number of technical complications. Patients were quite satisfied, as reflected by a mean VAS score of 94.0 ± 7.2 points (range 0–100) and a OHIP-NL49 score of 10.8 (range 0–196).

**Conclusions:**

Single implant-supported fixed partial dentures with a mesial or distal cantilever can be a predictable treatment option in the posterior region, with stable peri-implant bone levels, minor technical complications, and very content patients. However, the prevalence of peri-implant mucositis and peri-implantitis was high.

**Trial registration:**

ISRCTN, ISRCTN79055740, Registered on March 14, 2021 – —Retrospectively registered.

## Background

In a systematic review including studies with a follow-up of at least 10 years, Howe et al. calculated a 10-year implant survival rate of 96.4% in partially dentate patients [1]. Patients’ expectations with respect to dental implant treatment are high and usually they are met [2]. Although many treatment options are thoroughly evaluated, it is acknowledged that new trends evolve and that there are still remaining questions which need to be answered [3]. An example of such a question is whether it is clinically feasible to provide a single implant with a crown extended with a cantilever unit in case of two missing teeth in the posterior region of the maxilla or mandible.

In case of two missing teeth, the first option would be to provide two single-tooth implant-supported crowns. However, there are situations in which the diastema is too narrow (the implants would be too close to each other or too close to neighboring teeth) or because not enough bone volume is present at one of the implant sites and a bone augmentation procedure is not an option for the patient [4] or because one of two inserted implants failed to osseointegrate. In addition, two single implants with crowns may render treatment not feasible for patients because of financial limitations. To possibly overcome the above-mentioned problems, one implant with one crown extended with a mesial or distal cantilever could be the solution. However, this kind of construction bears the risk of overloading the implant and the suprastructure which could lead to biological and technical complications [4].

Van Nimwegen et al. performed a systematic review on single implant-supported two-unit cantilever fixed partial dentures (FPDs) [5]. Five articles could be included in which the implant survival ranged from 97 to 100% over a period ranging from 1 to 4 years. They concluded that a two-unit cantilever FPD in the anterior region is a viable treatment option. However, they observed a high number of technical complications in the posterior region and hence advised against a two-unit cantilever FPD for replacement of (pre-)molars. Based on a consensus conference of the European Association for Osseointegration, a systematic review on cantilever FPDs was published by Storelli et al. [6]. Only one study fulfilled their inclusion criteria: at least ten patients with a single implant-supported two-unit FPD in the posterior region that had been followed up for at least 5 years [4]. The average follow-up period of this particular retrospective study was 6.5 years. Implant survival was 100%. There was very little bone loss during the evaluation period. No technical complications were observed. Due to the very limited number of suitable studies, Storelli et al. stated that no conclusion could be drawn concerning the applicability of this treatment option, underscoring and emphasizing the lack of evidence for this treatment modality. No study ever reported on patient satisfaction about this treatment to the best of our knowledge. Therefore, the aim of the present retrospective study was to evaluate survival, clinical performance, complications, and patient-reported outcomes of single implant-supported two-unit cantilever FPD in the posterior region.

## Methods

The design of the study is a retrospective analysis of all patients treated with a single implant-supported two-unit cantilever FPD in the posterior region of the maxilla or mandible between the period January 1, 2004, and January 1, 2018, in a private referral practice in Apeldoorn, The Netherlands. Inclusion criteria for the study were:

- One dental implant restored with a crown with one cantilever unit positioned mesially or distally, in the posterior region of the maxilla or mandible
- Presence of antagonistic teeth
- Follow-up period at least 1 year after placement of the restoration
- Presence of a radiograph taken directly after placement of the implant

Patients fulfilling all the inclusion criteria were informed verbally and in writing about the study and signed the informed consent form. Two-unit cantilever fixed partial denture implant treatment in the posterior region was judged as being standard care in daily dental practice. The Medical Ethical Committee of the University Medical Center Groningen considered this retrospective case series study not to be subject to the Medical Research Involving Human Subjects Act (METc RR-Number 201800656).

### Surgical and prosthetic protocol

All patients were treated following the same treatment protocol by one surgeon (FLG). Implant surgery was performed using the standard Astra Tech Implant System protocol (document 79254-usx-1002 Astra Tech) to a final drill diameter. One hour preoperative antibiotic prophylaxis (3 g amoxicillin or, if allergic to penicillin, 600 mg clindamycin) was administered. Postoperative treatment included a chlorhexidine rinse twice daily starting 1 day before the operation and ending 10 days later. No further anti-microbial therapies were used. The surgical procedure was performed under local anesthesia. A crestal incision and performance of buccal and palatal flaps were made. Dependent on the height of the bone, an 8- to 13-mm implant (Astra OsseoSpeed™, Dentsply Implants, Mölndal, Sweden) was placed. The diameter of all implants was 4.0 mm. Maximum torque used during implant installation was set according to Astra Tech’s surgical manual, and primary implant stability was estimated manually. A cover screw was placed and the wound was closed with slowly resorbable sutures (Vincyl & Johnson Health Care, Piscataway, New Jersey). During the 12-week healing period, the implants were left in a submucosal position. One week after implant placement, a follow-up visit was scheduled for suture removal and review of the healing process. Twelve weeks after implant placement, second stage surgery was performed and a healing abutment was placed. Implant stability was manually examined.

**Fig. 1:**
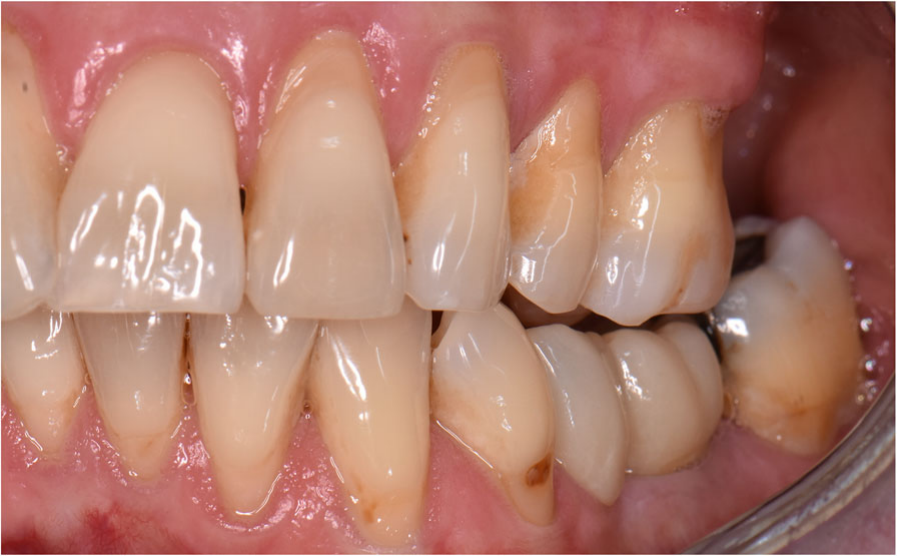
Clinical case of a cantilevered 2-unit FDP (lateral view). The implant is placed in position 36, the cantilever pontic in position 35

An impression at implant level was made 2 weeks after second stage surgery for fabrication of the FPD. Two weeks thereafter, either a titanium individual abutment (Atlantis Abutment, Dentsply Implants, Mölndal, Sweden) was placed (25Ncm torque) and a metal- or zirconia-based porcelain FPD with cantilever was cemented (GC Fuji 1, GC Europe NV, Leuven, Belgium) or a metal- or zirconia-based porcelain FPD with cantilever was directly screw-retained to the implant (25 Ncm torque) (Fig. 1).

### Outcome measures

#### Survival rate

Survival rate was defined as the percentage functional implants at the follow-up evaluation. The criteria for successful osseointegration according to Smith and Zarb were adopted [7]. Patients not examined at the follow-up evaluation were counted as having functional implants, unless their medical record revealed otherwise.

#### Radiographic assessments

To calculate changes in marginal bone level, a digital peri-apical radiograph was taken using a paralleling technique with an x-ray holder, immediately following implant placement (baseline) and at the follow-up evaluation.

**Fig. 2:**
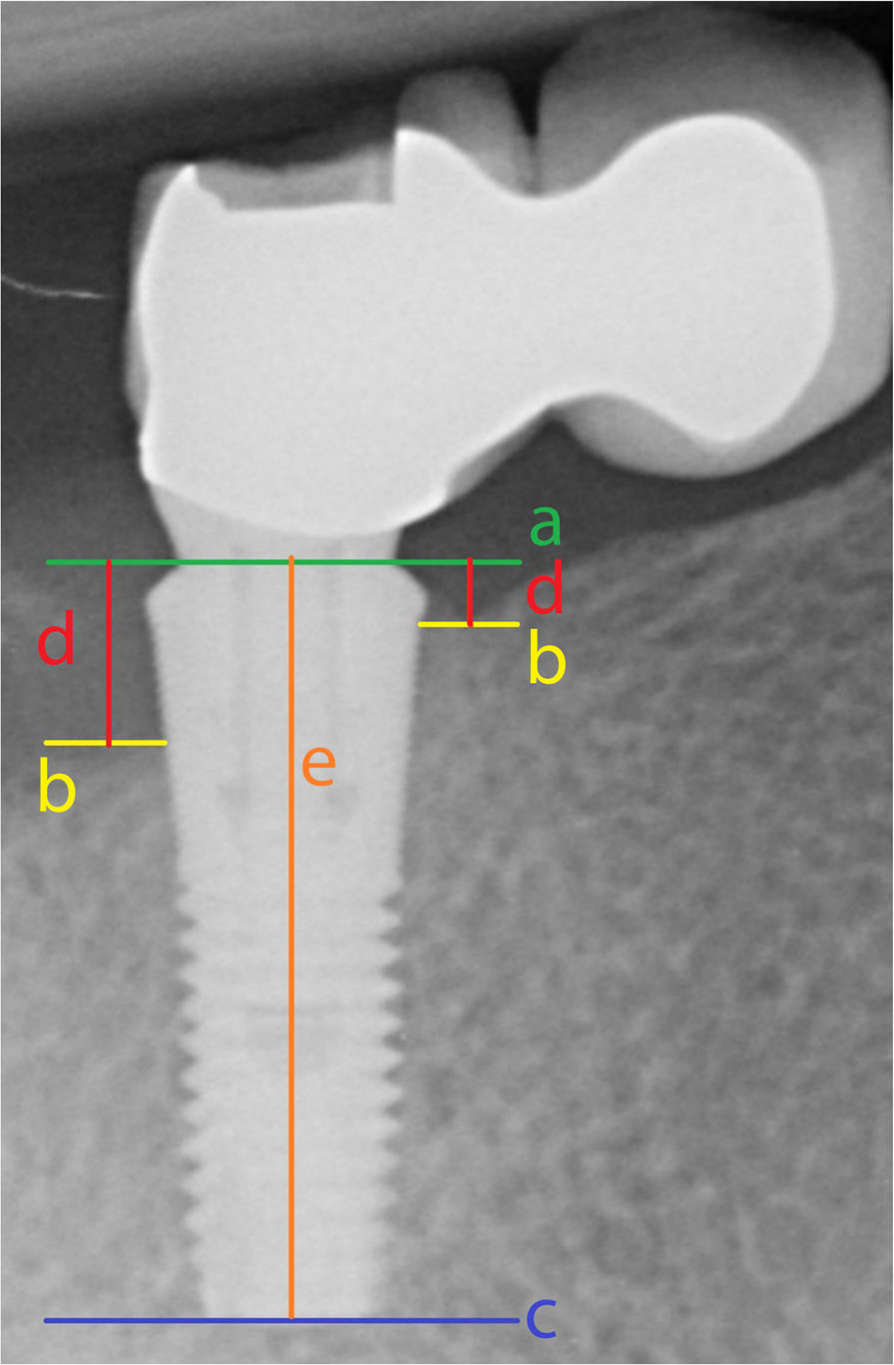
Measurement of marginal bone level at the mesial and distal side of the implant: Line a, reference line at interface of implant and abutment; line c, line at the apex of the implant; line e, length of the implant for calibration; line b, line through the first bone-to implant contact; line d, measurement of marginal bone level from reference line a to bone-to-implant contact line b

Radiographs were analyzed using the known implant length as a reference. The interface of the implant and the abutment was used as a reference line, from which all distances were measured with the designated software (DicomWorks, Biomedical Engineering, University Medical Center Groningen, The Netherlands). The error of this method was reported 0.13 ± 0.01 mm [8]. The following linear measurements were assessed to the nearest 0.01 mm: the vertical distance between the reference line and the first bone to implant level, measured at the mesial and distal side of the implant (Fig. 2). Measurements were performed by one examiner (HS).

#### Clinical assessments

The following clinical variables were assessed at the follow-up evaluation:

- Plaque: assessed per implant using the modified plaque index [9]
- Presence of calculus
- Bleeding: assessed per implant using the modified sulcus bleeding index [9]
- Gingival health: assessed per implant using the gingival index [10]
- Probing depth: assessed at four sites per implant using a manual standardized pressure periodontal probe (Click-Probe®, Kerr, Bioggio, Switzerland) measuring to the nearest 1 mm

All clinical data were retrieved by one examiner (HS).

#### Complications

Peri-implant mucositis and peri-implantitis were calculated at the implant level. As definition for peri-implant mucositis and peri-implantitis, the consensus reached at the Seventh European Workshop on Periodontology was used [11], being peri-implant mucositis (radiographic bone loss less than 2 mm), bleeding on probing and/or suppuration and peri-implantitis, and bleeding on probing and/or suppuration in combination with marginal bone loss of 2 mm or more.

All technical complications (e.g., restoration failure, cement loosening, screw loosening, fracture of veneering ceramics) throughout the period from restoration placement until the follow-up evaluation were noted at the follow-up evaluation and collected from the patients’ medical record.

#### Patients’ satisfaction

Patient satisfaction was measured using a self-administered questionnaire on the specific treatment outcome with a visual analogue scale (VAS; five questions) and the validated oral health-related quality of life questionnaire (Dutch-validated version of the Oral Health Impact Profile questionnaire: OHIP-NL49; 49 questions in 7 domains) [12, 13].

### Statistical analysis

All analyses were performed at implant level and restoration level, except for patients’ satisfaction. Descriptive statistics were applied to describe the means (with standard deviations) and medians (with interquartile ranges) of variables used in current study. For the bone level, in case of a normal distribution of data, a “one sample t-test” was used, if not, a non-parametric tests was used: the “Wilcoxon’s signed Rank test.”

Possible correlations between peri-implant bone level change and position of the implant (premolar/molar), jaw (maxilla/mandible), and method of attachment of the restoration (screw-retained/cement-retained) were calculated either with the “independent samples t-test” (sufficiently normal distribution) or with the “Mann-Whitney U test” (insufficiently normal distribution). Data were analyzed using the Statistical Package for Social Sciences (version 24.0, SPSS Inc., Chicago, Illinois).

## Results

Between January 1, 2004, and January 1, 2018, all consecutive patients (n = 35) who received a single implant-supported two-unit cantilever FPD in the posterior region of the maxilla or mandible had their medical records examined. Two patients had died (n = 2). Thirty-three patients were selected for clinical examination and invited by letter and telephone call to attend the clinic for a recall visit. Four patients moved without leaving a forwarding address (n = 4). Two patients were unable to attend due to the travel distance to the dental practice (n = 2) and four patients were unable to attend due to advanced age or sickness (n = 4). Twenty-three patients (n = 23) with altogether 28 single implant-supported two-unit cantilever FPDs in the posterior region of maxilla or mandible were able and willing to participate in the study. The assumption was made that not attending the follow-up evaluation was independent of possible complications or patient satisfaction. Patient and treatment characteristics of the study group are depicted in Table 1. The mean follow-up period was 6.5 ± 4.8 years at the time of data collection. The distribution of years at risk was as follows: 8 FDPs for 1 year, 3 FDPs for 2 years, 3 FDPs for 3 years, 1 FDP for 4 years, 1 FDP for 9 years, 5 FDPs for 10 years, 3 FDPs for 11 years, 3 FDPs for 12 years, and 1 FDP for 14 years.

**Table 1:** Patient and treatment characteristics

Number of patients/implants	23/28
Mean age at time of implant placement in years (SD/min.–max.)	64 (13/19-84)
Gender (male/female)	11/12
Maxilla/mandible	15/13
Implant position (premolar/molar)	12/16
Mesial cantilever/distal cantilever	23/5
Screw-retained/cement-retained	19/9
Porcelain-veneered zirconia/porcelain-veneered metal	14/14

Survival rate of implants and restorations was 100%. Mean marginal bone loss for the mesial and distal side of the implants was respectively 0.41 mm (SD 1.18 mm) and 0.63 mm (SD 0.98 mm) (Table 2). Table 3 shows a mean probing depth of 3.1 ± 1.3 mm around the implants.

**Table 2:** Mean marginal peri-implant bone level (mm) and mean marginal peri-implant bone level change (mm)

	Mean marginal bone level mesial side (sd)	Mean marginal bone level distal side (sd)
At time of implant placement	0.36 (1.07)	0.41 (0.84)
At time of follow-up evaluation	0.78 (0.82)	1.03 (1.02)
Marginal bone-level change	−0.41 (1.18)	−0.63 (0.98)
Minimum	0.00	0.00
Maximum	−3.30	−2.90

**Table 3:** Median (IQR) of plaque index, bleeding index, presence of calculus, gingiva index, and mean (sd) of pocket probing depth at time of follow-up evaluation

Plaque index (possible score 0–3)	0 [0; 1]
Presence of calculus (possible score 0–1)	0 [0; 0]
Bleeding index (possible score 0–3)	1 [1; 2]
Gingiva index (possible score 0–3)	0 [0; 0.75]
Pocket probing depth (in millimeter)	3.1 (1.3)
Minimum	1.0
Maximum	7.0

Mann-Whitney U tests failed to demonstrate a statistically significant difference in the amount of peri-implant bone level change and the position of the implant (premolar/molar, mesial side: p = 0.84; distal side: p = 0.64), the jaw (maxilla/mandible, mesial side: p = 0.82; distal side: p = 0.07), and attachment type (screw-retained/cement-retained, mesial side: p = 0.13; distal side: p = 0.94). Plaque, calculus, and gingiva scores were low. Most of the implants had score 1 on the bleeding-index (isolated bleeding spots visible after probing). A high percentage of peri-implant-implant mucositis (89.3%) and peri-implantitis (17.9%) was found and only a limited number of technical complications was seen (Table 4). Patients were highly satisfied with a mean VAS score of 94.0 ± 7.2 points (Table 5). The mean total OHIP-score was low (10.8 on a possible maximum score of 196), and quite favorable (Table 6).

**Table 4:** Biological and technical complications during the follow-up period

**Biological complications**	
Implant failure	0%
Peri-implant mucositis	89.3%
Peri-implantitis	17.9%
**Technical complications**	
Restoration failure	0%
Cement loosening	3.6%
Screw loosening	3.6%
Fracture of veneering ceramics	7.1%

**Table 5:** Patient satisfaction questionnaire with VAS scores (range 0–100)

	Mean	Standard deviation	Minimum-maximum
How satisfied are you with the cantilever FPD	94.0	7.2	73–100
Does the cantilever FPD meet your expectations	96.1	6.1	73–100
Do you like the design of the cantilever FPD	93.9	8.2	63–100
Do you like the color of the cantilever FPD	93.6	8.1	70–100
Would you recommend the cantilever FPD to anyone else	90.7	20.8	0–100

*VAS* visual analogue scale, *FPD* fixed partial denture

**Table 6:** Mean sum scores (with standard deviation) of Oral Health Impact Profile questionnaire (OHIP-NL49) at time of follow-up evaluation

	Mean (standard deviation)
Functional limitation (max. score 36)	3.9 (2.8)
Physical pain (max. score 36)	3.8 (4.0)
Psychological discomfort (max. score 20)	1.3 (3.0)
Physical disability (max. score 36)	1.0 (1.5)
Psychological disability (max. score 24)	0.3 (0.9)
Social disability (max. score 20)	0.2 (0.6)
Handicap (max. score 24)	0.3 (0.8)
OHIP total (max. score 196)	10.8 (8.5)

## Discussion

The data from the present study indicate that a single implant with an implant-supported FPD that consists of a mesial or distal cantilever can be a predictable treatment option in the posterior region. No implants were lost during a mean follow-up period of 6.5 years, which is in line with the findings of others who reported implant survival rates of 97-100% over 5-12 years [14–16] for this particular type of restoration. Also, all FPDs were still in function. Stable peri-implant bone levels and minor technical complications were seen and patients were quite content. Hence, concerns regarding the detrimental loading condition of the implants in terms of magnitude and direction proved unjustified, even though in literature a relationship between excessive or unfavorable loading of implants and (late) failures due to peri-implant bone loss has been proposed [17–19].

The amount of marginal bone loss (MBL, 0.41 mm and 0.63 mm) is small, and in agreement with the findings of Romeo and co-workers, who observed MBL of 0.8–1.2 mm around cantilevered prostheses in partially dentate jaws after a mean follow-up period of 8.4 years [14].

The position of the implant did not statistically significantly influence the amount of MBL. It is noteworthy that only 5 out of 28 FPDs had a cantilever on the distal end of the implant. Rossi et al. reported more bone loss around implants placed in the maxilla compared to the mandible after a follow-up period of 10 years [20], but in the present study focusing on a particular type of construction, such difference could not be demonstrated. The same counts for the fixation type that was applied (screw- or cement-retained). In general, reports on this matter are ambiguous: some state that screw-retained restorations are associated with less marginal bone loss, where others have seen the opposite [21, 22].

The high prevalences of peri-implant mucositis (89.3%) and peri-implantitis (17.9%) have not led to implant failure or excessive marginal bone loss during the observation period, but are ground for concern on the long run. The paradox of having a high percentage of implants with peri-implantitis and a rather low mean peri-implant bone level change (0.41 mm at the mesial side and 0.63 mm at the distal side) can be explained by the fact that even though a number of implants had more than 2 mm of bone loss during the evaluation period, the vast majority had no bone loss or very limited bone loss. For this retrospective study, all subjects who were treated with a single implant-supported two-unit cantilever FPDs in the posterior region were included, regardless of their general health or smoking habits, which could be of influence [23]. Another possible explanation could be the retrospective nature of the study. The subjects were not subjected to a strict oral hygiene regime, as is mostly the case in prospective studies.

Some technical complications were seen, with porcelain chipping being the most prevalent problem. They could all be managed rather easily. Van Nimwegen et al. reported a higher percentage of technical complications in single implant-supported two-unit cantilever FPD in the posterior region compared to the anterior region [5]. The larger lever arm and higher forces evidently put more strain on constructions in the dorsal areas of the jaw compared to those more to the anterior. Strain is directed from the point of engagement, through the connection of the pontic and the adjacent crown to the implant [24]. It induces internal stresses and strains which can lead to chipping or fracture of (a part of) the construction or the implant itself. This is also confirmed by Hälg et al., who studied implant-supported 2-unit cantilevers in the (pre-)molar region: more technical complications occurred when compared to two adjacent implant-supported single crowns [25]. Screw loosening in some cases is also seen by others [26]. Despite the risk of technical complications, several authors concluded that single implant-supported two-unit cantilever FPDs provide a viable treatment option when there is only limited bone volume to place two adjacent implants or in order to save money [6, 27]. The present authors confirm this.

Patients were quite content as reflected by favorable VAS and OHIP scores. There is no literature available on patient satisfaction to compare these data with.

Jung et al. [28] reported in a systematic review with meta-analysis on 46 included studies 5-year results of single implants with single implant-supported crowns without a cantilever. They reported a 5-year implant survival of 97.2% and a 5-year crown survival of 96.3%. In the present study, implant survival and restoration survival were both 100%. The fact that a crown is extended with a cantilever seems not to have an influence on survival of either the implant or the restoration.

The present study has limitations; its retrospective nature being the most prominent one, the absence of a control group consisting of cases with two single implants is another one. The group size was rather small and not all subjects were able to attend the control visit. Hence, some assumptions had to be made. The data were retrieved from a single practice, treatment being performed by an experienced surgeon and prosthodontist, using one particular implant system. This limits the external validity.

## Conclusions

Single implant-supported FPDs with a mesial or distal cantilever can be a predictable treatment option in the posterior region, with stable peri-implant bone levels, minor technical complications, and a high patient-satisfaction. However, the prevalence of peri-implant mucositis and peri-implantitis was high.

## Data Availability

The datasets used and/or analyzed during the current study are available from the corresponding author on reasonable request.

## Abbreviations

FPD: Fixed partial denture
MBL: Marginal bone loss
OHIP: Oral health impact profile
VAS: Visual analogue scale

## References

[1] Howe MS, Keys W, Richards D (2019). Long-term (10-year) dental implant survival: a systematic review and sensitivity meta-analysis. J Dent.

[2] Korfage A, Raghoebar GM, Meijer HJA, Vissink A (2018). Patients’ expectations of oral implants: a systematic review. Eur J Oral Implantol.

[3] Buser D, Sennerby L, De Bruyn H (2017). Modern implant dentistry based on osseointegration: 50 years of progress, current trends and open questions. Periodontol 2000.

[4] Aglietta M, Iorio Siciliano V, Blasi A, Sculean A, Brägger U, Lang NP, Salvi GE (2012). Clinical and radiographic changes at implants supporting single-unit crowns (SCs) and fixed dental prostheses (FDPs) with one cantilever extension. A retrospective study. Clin Oral Implants Res.

[5] Van Nimwegen WG, Raghoebar GM, Tymstra N, Vissink A, Meijer HJA (2017). How to treat two adjacent missing teeth with dental implants. A systematic review on single implant-supported two-unit cantilever FDP’s and results of a 5-year prospective comparative study in the aesthetic zone. J Oral Rehabil.

[6] Storelli S, Del Fabbro M, Scanferla M, Palandrani G, Romeo E (2018). Implant supported cantilevered fixed dental rehabilitations in partially edentulous patients: systematic review of the literature. Part I. Clin Oral Implants Res.

[7] Smith DE, Zarb GA (1989). Criteria for success of osseointegrated endosseous implants. J Prosthet Dent.

[8] Meijndert L, Meijer HJ, Raghoebar GM, Vissink A (2004). A technique for standardized evaluation of soft and hard peri-implant tissues in partially edentulous patients. J Periodontol.

[9] Mombelli A, Van Oosten MA, Schiirch E, Lang NP (1987). The microbiota associated with successful or failing osseointegrated titanium implants. Oral Microbiol Immunol.

[10] Loë H, Silness J (1963). Periodontal disease in pregnancy. I. Prevalence and severity. Acta Odontol Scand.

[11] Lang NP, Berglundh T, Working Group 4 of Seventh European Workshop on Periodontology (2011). Periimplant diseases: where are we now?--Consensus of the Seventh European Workshop on Periodontology. J Clin Periodontol.

[12] Slade GD, Spencer AJ (1994). Development and evaluation of the Oral Health Impact Profile. Community Dent Health.

[13] Van der Meulen MJ, Lobbezoo F, John MT, Naeije M (2011). Oral health impact profile. an instrument for measuring the impact of oral health on the quality of life. Ned Tijdschr Tandheelkd.

[14] Romeo E, Tomasi C, Finini I, Casentini P, Lops D (2009). Implant-supported fixed cantilever prosthesis in partially edentulous jaws: a cohort prospective study. Clin Oral Implants Res.

[15] Malo P, de Araujo NM, Lopes A (2013). The prognosis of partial implant-supported fixed dental prostheses with cantilevers. A 5-year retrospective cohort study. Eur J Oral Implantol.

[16] Roccuzzo A, Jensen SS, Worsaae N, Gotfredsen K (2020). Implant-supported 2-unit cantilevers compared with single crowns on adjacent implants: a comparative retrospective case series. J Prosthet Dent.

[17] Quirynen M, Naert I, van Steenberghe D (1992). Fixture design and overload influence marginal bone loss and fixture success in the Branemark system. Clin Oral Implants Res.

[18] van Steenberghe D, Naert I, Jacobs R, Quirynen M (1999). Influence of inflammatory reactions vs. occlusal loading on peri-implant marginal bone level. Adv Dent Res.

[19] Coli P, Christiaens V, Sennerby L, Bruyn H (2017). Reliability of periodontal diagnostic tools for monitoring peri-implant health and disease. Periodontol 2000.

[20] Rossi F, Lang NP, Ricci E, Ferraioli L, Baldi N, Botticelli D (2018). Long-term follow-up of single crowns supported by short, moderately rough implants-a prospective 10-year cohort study. Clin Oral Implants Res.

[21] Amorfini L, Storelli S, Mosca D, Scanferla M, Romeo E (2018). Comparison of cemented vs screw-retained, customized computer-aided design/computer-assisted manufacture zirconia abutments for esthetically located single-tooth implants: a 10-year randomized prospective study. Int J Prosthodont.

[22] Sailer I, Muhlemann S, Zwahlen M, Hammerle CH, Schneider D (2012). Cemented and screw-retained implant reconstructions: a systematic review of the survival and complication rates. Clin Oral Implants Res.

[23] French D, Grandin HM, Ofec R (2019). Retrospective cohort study of 4,591 dental implants: analysis of risk indicators for bone loss and prevalence of peri-implant mucositis and peri-implantitis. J Periodontol.

[24] Tribst JPM, Dal Piva AMO, Bottino MA, Nishioka RS, Borges ALS, Ozcan M (2020). Digital image correlation and finite element analysis of bone strain generated by implant-retained cantilever fixed prosthesis. Eur J Prosthodont Restor Dent.

[25] Halg GA, Schmid J, Hammerle CH (2008). Bone level changes at implants supporting crowns or fixed partial dentures with or without cantilevers. Clin Oral Implants Res.

[26] Palmer RM, Howe LC, Palmer PJ, Wilson R (2012). A prospective clinical trial of single Astra Tech 4.0 or 5.0 diameter implants used to support two-unit cantilever bridges: results after 3 years. Clin Oral Implants Res.

[27] Pjetursson BE, Brägger U, Lang NP, Zwahlen M (2007). Comparison of survival and complication rates of tooth-supported fixed dental prostheses (FDPs) and implant-supported FDPs and single crowns (SCs). Clin Oral Implants Res.

[28] Jung RE, Zembic A, Pjetursson BE, Zwahlen M, Thoma DS (2012). Systematic review of the survival rate and the incidence of biological, technical, and aesthetic complications of single crowns on implants reported in longitudinal studies with a mean follow-up of 5 years. Clin Oral Implants Res.

